# Point-of-care diagnosis and risk factors of infantile, rotavirus-associated diarrhoea in Calabar, Nigeria

**DOI:** 10.4102/ajlm.v6i1.631

**Published:** 2017-12-08

**Authors:** Samuel E. Nnukwu, Simon J. Utsalo, Olufunmilayo G. Oyero, Michel Ntemgwa, James A. Ayukekbong

**Affiliations:** 1 Department of Medical Laboratory Science, Faculty of Allied Medical Sciences, University of Calabar, Calabar, Cross River State, Nigeria; 2 Institute for Advanced Medical Research and Training, College of Medicine, University of Ibadan, Ibadan, Oyo State, Nigeria; 3 Health Products and Food Branch, Health Canada, Ottawa, Ontario, Canada; 4 Section for Clinical Virology, Redeem Biomedical, Buea, South West Region, Cameroon; 5 Metabiota, Nanaimo, British Columbia, Canada

## Abstract

**Background:**

Rotaviruses are the primary cause of acute gastroenteritis in young children worldwide and a significant proportion of these infections occur in Africa.

**Objectives:**

In the present study, we determined the prevalence and risk factors of rotavirus infection among children younger than age 5 years with or without diarrhoea in Calabar, Nigeria, using a rapid point-of-care test.

**Methods:**

Two hundred infants younger than age 5 years presenting with acute gastroenteritis and a control group of 200 infants without diarrhoea were tested for rotavirus. Each stool sample was homogenised in an extraction buffer and the supernatant added into the sample well of the Rida Quick rotavirus test cassette and allowed to run for 5 minutes at room temperature. When both the control band and test band were visible on the test cassette a positive result was recorded, whereas when only the control band was visible a negative results was recorded.

**Results:**

Rotavirus was detected in 25 (12.5%) of children with diarrhoea and in no children without diarrhoea. Our results demonstrated that children who were exclusively breast-fed by their mothers were not infected with rotavirus and that 92% of the infants infected with rotavirus experienced vomiting.

**Conclusion:**

These data demonstrate that asymptomatic rotavirus infection is rare and that rotavirus is commonly detected in stool samples of children suffering from diarrhoea with concomitant vomiting. Use of point-of-care rotavirus tests will enhance early diagnosis of rotavirus-associated diarrhoea and reduce irrational use of antibiotics.

## Introduction

Diarrhoea is a major cause of infantile morbidity and mortality in developing countries and it is increasingly recognised as a disease of poverty.^1^ Annually, more than 1 billion episodes of diarrhoea occur among children younger than age 5 years resulting in about 2.5 million deaths.^1,2^ More than 40% of global diarrhoea-associated deaths occur in Africa;^3,4^ the risk of contracting diarrhoeal diseases has been suggested to be higher in developing countries compared to developed countries, due in part to unsafe water supplies, sub-optimal sanitation and unhygienic conditions.^5^ Diarrhoea is a manifestation of intestinal dysfunction that causes frequent watery stool, resulting in loss of water, electrolytes and nutrients.^6^

The infectious cause of diarrhoea is multifactorial, with viruses, bacteria, protozoans or helminths often involved.^7,8,9^ In developing-country settings, microscopy (for the detection of protozoa and the ova of helminths) and bacteria culture are routinely performed to determine the cause of diarrhoea. Virus investigation is often neglected. Acute infantile diarrhoea has been shown to be commonly caused by viruses, notably group A rotaviruses, which are transmitted primarily via the faecal-oral route.^10^ The incubation period is usually between 2 and 6 days and symptoms may last for up to 5–7 days.^11,12^ Rotavirus illness usually starts with an acute onset of fever and vomiting, followed by mild watery diarrhoea to frequently profuse diarrhoea that can result in severe dehydration, electrolyte imbalance and death.^13,14^ By age 5 years, nearly all children have been infected with rotavirus at least once,^11^ with severe infections occurring between age 6 months and 2 years, particularly in immunocompromised children.^15,16^ To date, there are two oral rotavirus vaccines: a pentavalent bovine human reassortment vaccine (RV5; Rotateq, Merck, New Jersey, United States) and a monovalent (G1P8 attenuated human rotavirus vaccine RV1; Rotarix, GlaxoSmithKline Biologicals, Belgium).^15,17,18^ However, there is limited access to routine vaccination, especially in rural communities, coupled with the fact that testing of rotavirus is often neglected in routine medical practice in Nigeria. Contrary to therapeutic guidelines, most diarrhoea cases are treated with antibiotics or anti-parasitic drugs and the contribution of rotavirus to diarrhoeal disease is widely neglected in routine clinical practice.

Globally, molecular diagnostic methods such as real-time polymerase chain reaction and enzyme-linked immunosorbent assay (ELISA) have improved the detection of rotavirus over the years.^19,20^ However, the routine use of these assays in developing countries is limited due to the cost and skill required to perform the analysis.^21^ The Rida Quick rotavirus assay is a useful alternative to polymerase chain reaction and ELISA.^22^ This assay is cheap (< $2 per test) and easy to perform, provides results within 5 min and does not require the use of sophisticated equipment or skilled training. Compared to polymerase chain reaction, the specificity of this assay has also been evaluated to be 95% and the sensitivity to be 100%.^22^

The aim of this study was to determine the prevalence and risk factors of rotavirus infection among children younger than age 5 years with or without diarrhoea in Calabar, Nigeria, using a rapid point-of-care test.

## Methods

### Ethical considerations

Ethical clearance was obtained from the Cross River State Ministry of Health ethics committee (RP/REC/2015/108) and participants (parents or guardians of children) provided written or oral inform consent.

### Study design

We conducted a cross-sectional study on the prevalence of rotavirus among children younger than age 5 years with diarrhoea and without diarrhoea in Calabar, Nigeria, from August to December 2015. Sample size was calculated based on a retrospective estimate of the number of admissions due to diarrhoeal disease in children younger than age 5 years at the selected sites (calculated standard deviation was 25), with a 95% confidence interval and a 5% margin of error. Two hundred infants younger than age 5 years presenting with acute gastroenteritis at four health establishments in Calabar were tested. A control group of 200 infants without diarrhoea was also included. Data on breastfeeding, hygiene practices, daycare/nursery school enrolment, drinking water sources were collected through questionnaires by trained personnel in a designated private area within the hospitals.

### Sample collection, processing and test procedure

**FIGURE 1:**
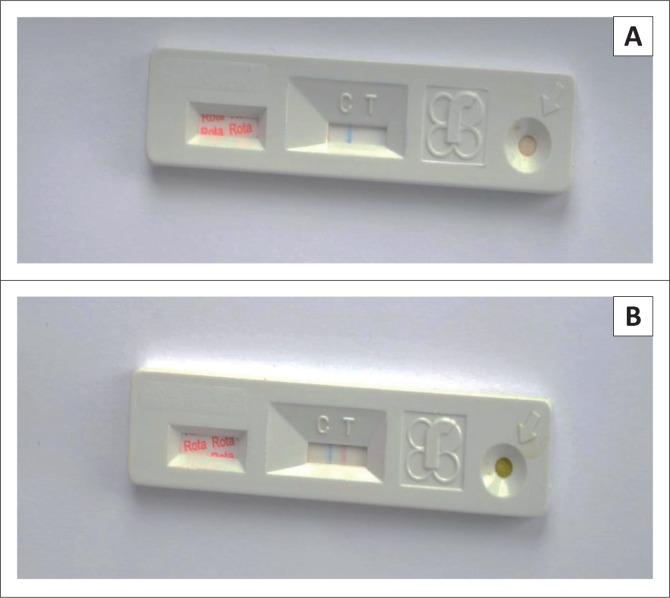
Rida Quick rotavirus Combi test cassette (R-Biopharm AG, Darmstadt, Germany) showing negative (A) and positive (B) results for rotavirus.

Two hundred stool samples were collected by trained health workers in sterile, leak-proof containers from infants younger than age 5 years with acute diarrhoea at the sentinel hospitals. In parallel, 200 stool specimens were collected from a control group of infants younger than age 5 years without diarrhoea. These controls were matched for age, gender and/or enrolment location. About 50 mg of stool sample was collected from the stool container using a sterile applicator and transferred into a sample tube containing 1 ml of extraction buffer and mixed gently to make a homogeneous mixture. About 2 to 3 drops of the supernatant from the homogeneous were was added into the sample well of the Rida Quick rotavirus Combi test cassette (R-Biopharm AG, Darmstadt, Germany) and allowed to run for 5 min at room temperature. When both the control band and test band were visible on the test cassette a positive result was recorded, whereas when only the control band was visible a negative result was recorded (Figure 1).

### Statistical comparison

A two-sided Fisher’s test was used to compare proportions with alpha set at 0.05. Odd ratios and 95% confidence intervals were calculated using the SPSS software package v. 17.0 for Mac (SPSS Inc., Chicago, Illinois, United States).

## Results

### Demographic characteristics of participants

Amongst the 200 cases, 56.5% (113/200) were male and 43.5% (87/200) female (Table 1). Children younger than age 2 years constituted 61% of the study population, and the overall mean age was 22 months (range: 3–44 months). In the control group of 200 children younger than age 5 years, the mean age was 25 months (range: 3–47 months). Male children constituted 55% (110/200) of the control group.

**TABLE 1:** Demographic characteristics and rotavirus prevalence among children with and without diarrhoea in Calabar, Nigeria, August to December 2015.

Characteristic	All participants *n*^a^	Rotavirus infection in children with diarrhoea *n* (%)	Rotavirus infection in children without diarrhoea *n* (%)
Age (months)			
< 6	8	1 (12.5)	0 (0)
7–12	43	13 (30.2)	0 (0)
13–24	71	8 (11.3)	0 (0)
25–34	51	2 (3.9)	0 (0)
35–60	27	1 (3.7)	0 (0)
Total	200	25 (12.5)	0 (0)
Gender			
Male	113	15 (13.3)	0 (0)
Female	87	10 (11.5)	0 (0)
Total	200	25 (12.5)	0 (0)
Study centres			
UCTH	119	16 (13.4)	0 (0)
General hospital	28	5 (17.9)	0 (0)
PHC Ekpo Abasi	31	3 (9.7)	0 (0)
PHC Ikot Ansa	22	1 (4.5)	0 (0)
Total	200	25 (12.5)	0 (0)

Abbreviations: UCTH, University of Calabar Teaching Hospital; PHC, Primary Health Centre.

a Cases and controls; the sub-population of children with diarrhoea was the same as those without diarrhoea

### Rotavirus detection by Rida Quick Combi immunochromatographic test

Rotavirus was detected in 25/200 (12.5%) of the children with acute gastroenteritis, with no difference between male and female patients (15/113, 13.3% vs 10/87, 11.5% *p* = 0.1) (Table 1). However, the prevalence was higher among children between ages 7 and 12 months (13/43; 30.2%) compared with other age groups (Table 1). A total of 23 of the 25 children (92%) diagnosed with rotavirus infection reported vomiting (data not shown). In terms of study centre, both the University of Calabar Teaching Hospital and the general hospital had a high prevalence of rotavirus infection compared with the Abasi and Ansa primary healthcare centres. Meanwhile, rotavirus was not detected in any of the 200 control children without diarrhoea for the same age ranges.

### Risk factors for rotavirus infection

In order to investigate the relationship between feeding method of children and the occurrence of rotavirus, we compared the prevalence of rotavirus infection among children who were exclusively breastfed to those who were not exclusively breastfed (that is, children in this category received breast milk plus solid food or solid food only). The results showed that no rotavirus infection was detected among children who were exclusively breastfed, whereas a 12.9% rotavirus prevalence was observed in children who were not (Table 2). There was also a statistically significant difference in the prevalence of rotavirus among children who attended nursery schools (21/99, 21.4%) compared with those who were not enrolled in any institution (4/101, 3.9%; *p* = 0.0001). We also investigated the relationship between hand washing before meals and the source of drinking water and the risk of rotavirus infection in children younger than age 5 years. There was no statistical difference in rotavirus prevalence among children who washed hands either independently or with the help of their parents or guardian before meals (20/161, 12.4%) compared with those who did not (5/39, 12.8%; *p* = 0.5). There was no statistically significant difference in rotavirus prevalence based on the source of drinking water.

**TABLE 2:** Risk factors of rotavirus infection among children younger than age 5 years in Calabar, Nigeria, August to December 2015.

Variable	Total	Rotavirus-infected cases *n* (%)	Odd ratio (95% confidence interval)	*p*-value
Breastfeeding practice				
Exclusive	7	0 (0)	0.14^a^	0.5
Non-exclusive^a^	193	25 (12.9)		
Hygiene practice				
Hand washing before meals^b^	161	20 (12.4)	0.96 (0.3–2.7)	0.5
No hand washing before meals	39	5 (12.8)		
Enrolment in institution				
Attend day care/nursery	99	21 (21.4)	6.6 (2.1–20.0)	0.0001
Does not attend day care/nursery	101	4 (3.9)		
Source of drinking water				
Bottled water	51	8 (15.6)	1.4 (0.5–3.5)	0.4
Tap water	88	10 (11.4)		
Borehole well water	61	7 (11.4)		

a Children who consumed breast milk and solid food or solid food only. The number of breastfed infants was small, OR determination was difficult.

b Hand washing was either done by the children or by their guardian or parents.

## Discussion

In this cross-sectional study, a rapid immunochromatographic point-of-care rotavirus detection assay was used to investigate the prevalence and risk factors of rotavirus infection among children younger than age 5 years in south-eastern Nigeria. The prevalence of 12.5% for rotavirus among children with diarrhoea in this study is consistent with that of another study in Nigeria where a prevalence of 13.8% was observed.^23^ This study also revealed that there was no statistically significant difference in the prevalence of rotavirus infection between male children and female children which is consistent with a previous report.^23^ A striking finding was the fact that no child who was exclusively breastfed tested positive for rotavirus. This corroborates evidence that breast milk may offer specific protection against rotavirus by the ‘decoy’ action of human milk glycans.^24,25^ This finding is compatible with the work of Quigley et al.,^26^ who stated that breastfed babies are four times less likely to experience diarrhoea associated with rotavirus than bottle-fed babies. Also, the highest prevalence of rotavirus infection was observed in children between age 7 and 12 months, a period that coincides with the weaning period from breastfeeding of most infants. This further corroborates the protective effect of breast milk on rotavirus infection. This observation is also consistent with studies that suggest that most severe rotavirus infections occur between ages 6 months and 2 years.^27,28^

We observed that the act of washing hands before meals did not confer any protection against rotavirus infection in the children studied. Also, children who attended day care were more prone to rotavirus infection. This is consistent with previous findings where outbreaks of rotavirus infection have been more commonly reported from day care centres and nursery schools.^27^ Overcrowding in most day care and nursery schools may facilitate transmission of rotavirus infection among children.

There was no difference in rotavirus infection among children who consumed different sources of water, such as tap water, borehole or well water, and bottled water. This finding is in contrast with our previous report where the risk of rotavirus infection was higher among consumers of water from boreholes or wells compared with consumers of tap water.^19^ The fact that rotavirus was not detected in children without diarrhoea suggests that asymptomatic rotavirus infection is rare. Also, 92% of children with rotavirus infection suffered from vomiting. This finding is consistent with previous scientific and clinical observations that diarrhoea and vomiting are the hallmarks of rotavirus infection.^13,29^ Although rotavirus was more prevalent in hospital settings compared with primary health centres, the results were not statistically significant. A plausible explanation is that due to the lack of local capacity in most primary health centres, serious health issues are referred to hospitals for proper management.

### Limitations

This study, although carefully conducted, was subject to some limitations. Perhaps the most compelling is that the study was limited to a small population in Calabar and thus may not reflect the general population. Secondly, because of lack of capacity, the investigators could not genotype the 25 rotavirus-positive samples, which could have conveyed further interesting findings on the epidemiology of the infection in the region. Thirdly, other diarrhoea-causing pathogens were not investigated; therefore, the cause of diarrhoeal episodes cannot solely be linked to rotavirus infection alone.

Despite these limitations, the study is the first of its kind in south-eastern Nigeria and suggests that asymptomatic rotavirus infection in children younger than age 5 years is rare. The study also provides additional knowledge on the risk factors of rotavirus infection and corroborates findings that exclusive breastfeeding may confer a protective advantage to infants under age 6 months against rotavirus infection. It also confirms the fact that day care and nursery school settings predispose children to rotavirus infection, as they are likely to be infected by other children.

### Conclusion

Taken together, this study suggests that routine use of a rapid immunochromatographic test may enhance early detection of rotavirus and guide clinical management. This will ultimately reduce the burden of the disease in children and prevent the irrational prescription of antibiotics.
